# Effects of Ultra-Low Temperatures on the Mechanical Properties and Microstructure Evolution of a Ni-Co-Based Superalloy Thin Sheet during Micro-Tensile Deformation

**DOI:** 10.3390/ma16216838

**Published:** 2023-10-24

**Authors:** Qiang Zhu, Min Wang, Yuying Sun, Linfu Zhang, Heyong Qin, Peng Zhang

**Affiliations:** 1 School of Materials Science and Engineering, Harbin Institute of Technology at Weihai, Weihai 264209, China; zhuqiang@hit.edu.cn (Q.Z.); 22b909134@stu.hit.edu.cn (M.W.); sunyuyingyys@163.com (Y.S.); 21b909090@stu.hit.edu.cn (L.Z.); 2 Key Laboratory of Micro-Systems and Micro-Structures Manufacturing of Ministry of Education, Harbin Institute of Technology, Harbin 150080, China; 3 High Temperature Materials Research Division, Central Iron & Steel Research Institute, Beijing 100081, China; qinheyong@126.com

**Keywords:** Ni-Co-based superalloy, size effect, mechanical properties, microstructure evolution

## Abstract

With the development of product miniaturization in aerospace, the nuclear industry, and other fields, Ni-Co-based superalloys with excellent overall properties have become key materials for micro components in these fields. In the microforming field, size effects significantly impact the mechanical properties and plastic deformation behavior of materials. In this paper, micro-tensile experiments at room temperature and an ultra-low temperature were carried out to study the effects of initial microstructure and deformation temperature on the deformation behavior of Ni-Co-based superalloy thin sheets. The results show that as the ratio of specimen thickness to grain size (*t*/*d*) decreased from 8.6 to 2.4, the tensile strength *σ*_b_ decreased from 1221 MPa to 1090 MPa, the yield strength *σ*_s_ decreased from 793 MPa to 622 MPa, and the elongation decreased from 0.26 to 0.21 at room temperature. When *t*/*d* decreased from 8.6 to 2.4, *σ*_b_ decreased from 1458 MPa to 1132 MPa, *σ*_s_ decreased from 917 MPa to 730 MPa, and the elongation decreased from 0.31 to 0.28 at ultra-low temperatures. When *t*/*d* decreased from 8.6 to 2.4, the surface roughness of the specimen increased from 0.769 to 0.890 at room temperature and increased from 0.648 to 0.809 at ultra-low temperatures. During the microplastic deformation process of Ni-Co-based superalloy thin sheets, the coupled effects of surface roughening caused by free surface grains and hindered dislocation movement induced by grain boundary resulted in strain localization, which caused fracture failure of Ni-Co-based superalloy thin sheets.

## 1. Introduction

Product miniaturization is one of the main trends in advanced manufacturing technology. Micro-electro-mechanical systems (MEMS) emerged under this situation. Microforming technology has the characteristics of large volumes, high efficiency, high precision, low costs, and no pollution and has become a key technology for the manufacturing of thin sheet microstructure parts [1,2,3]. Superalloys are indispensable key materials for the development of the modern aerospace industry, which are mainly used to manufacture turbine disks and other hot-end components of aerospace engines [4,5,6,7]. With the development of the aerospace industry, higher requirements have been placed on hot-end components. However, existing nickel-based superalloys no longer meet the requirements for higher service temperatures. Improving the strength of nickel-based superalloys without losing their plasticity has always been a focus of researchers. However, an inverted relationship between strength and plasticity occurs in nickel-based superalloys. Studies have shown that the inverted relationship between strength and plasticity can be broken by reducing stacking fault energy (SFE). The National Institute for Materials Science of Japan successfully developed the TMW series Ni-Co-based superalloys first by reducing SFE [8]. Compared with the stronger cast-forged U720Li superalloy, the temperature-bearing capacity of this Ni-Co-based superalloy can be increased by 30 °C. The mechanical properties and structural stability of Ni-Co-based superalloys are better than those of the U720Li superalloy, which is expected to become a key material for hot-end components [9,10,11,12]. With the unstoppable trend of product miniaturization in many fields such as aerospace, the nuclear industry, weapons equipment, and energy, precise plastic forming technology of Ni-Co-based superalloy micro components is a key task that needs to be solved urgently. Although traditional plastic forming technology and theory are well-established, microforming technology does not simply minimize the geometry of the specimen. As the size of the specimen decreases, it shows obvious size effects, causing its microplastic deformation behavior to be extremely complex [13,14].

To name a few, previous studies [15,16,17,18,19,20,21,22,23,24] have reported that the flow stress of thin sheets decreased with decreasing *t*/*d*, and a “smaller is weaker” size effect appeared. In the microforming of metal thin sheets, the strain coordination ability between grains decreased with decreasing *t*/*d*, resulting in a decrease in the homogeneous plastic deformation ability. Nie et al. [25] conducted micro-tensile tests on commercial pure titanium foils with thicknesses of 0.1 and 0.2 mm. They found that the micro-tensile yield strength and elongation of pure titanium thin sheets decreased with decreasing *t*/*d*. As *t*/*d* decreased, the fracture mechanism changed from ductile fracture to brittle fracture. Zhao et al. [26] conducted micro-tensile tests on Ti65 alloy foils with thicknesses of 2.0 mm, 0.5 mm, and 0.1 mm at room temperature. They found that silicide precipitation in the heat-treated specimens led to a more obvious “smaller is weaker” phenomenon and the specimens with transverse textures showed a better strength–ductility match. Zhu et al. [27] conducted high strain rate micro/meso-tensile tests on hot-rolled TA1 pure titanium foil with a thickness of 103 μm. They found that the size effect on flow stress was more and more obvious at the quasi-static tensile test while it became invariant when strain reached a specific value in the dynamic tensile test. Kang et al. [28] conducted a micro-tensile test on pure copper with a thickness of 0.2 mm to investigate the effect of simultaneous ultrasonic vibration during plastic deformation. They found that the flow stress reduction was more significant when vibration amplitude increased from 1.0 μm to 1.3 μm because ultrasonic vibrations promoted preferential grain re-orientation and reduced the internal misorientation within grains. Fu et al. [29,30] found that the fracture strain and the number of micropores on the fracture surface decreased with decreasing *t*/*d* for pure copper foils with thicknesses ranging from 100 to 600 μm.

Existing research shows that ultra-low temperatures play a very good strengthening and toughening role in face-centered cubic (FCC) metal [31,32,33]. The total elongation and strain hardening index of the aluminum alloy sheet at liquid nitrogen temperature were almost enhanced one time more than that at room temperature [34]. Rokilan and Mahendran [35] found that the reducing temperature increased the yield and ultimate strengths of high-strength cold-rolled steel G550 in a temperature range of 20–70 °C. Xi et al. [36] found that decreasing the low temperature from 20 to −165 °C significantly increased the yield and ultimate strengths of HSS Q690E and Q960E materials. Zang et al. [37] found that a breakthrough in the cryogenic ductility of titanium alloys was achieved, with elongation to fracture being up to 20.0% at 20 K and 29.0% at 77 K without significantly sacrificing strength. Levings and Sritharan [38] found that the yield strength and ultimate strength of ASTM A706 [39] Grade 420 steel increased by 5.1% and 6.3%, respectively, at −40 °C. Yan et al. [40] found that the yield strength, Young’s modulus, ultimate strength, and fracture strain increased with decreasing temperature. Techniques such as ultra-low temperature stamping [41,42], ultra-low temperature deep drawing [43], and ultra-low temperature gradient forming [44] have been implemented to improve the forming limit by enhancing ductility at ultra-low temperatures.

To date, research on size effects mainly focuses on room temperature and high temperatures. Ultra-low temperature forming is still a relatively new and potential forming method to improve the mechanical properties of metal materials. The deformation characteristics of materials at ultra-low temperatures are very different from those at room temperature, such as mechanical properties, microstructure evolution, fracture mechanism, etc. Therefore, this study carried out micro-tensile tests on Ni-Co-based superalloys at room temperature and an ultra-low temperature. The microstructural evolution behavior during micro-tensile deformation was characterized and analyzed using electron backscatter diffraction (EBSD) and scanning electron microscopy (SEM). The impacts of grain and ultra-low temperatures on the micro-tensile properties and deformation mechanism of Ni-Co-based superalloy thin sheets are clarified. This study provides a theoretical basis for the forming process of Ni-Co-based superalloy thin sheet micro components and has practical application value in promoting its application in the microforming field.

## 2. Materials and Methods

Cold-rolled and annealed Ni-Co-based superalloy thin sheets were selected as the experimental material. The main mass percentages of Ni-Co-based superalloys were 25% Co, 10% Cr, 4% Mo, 1% W, 2% Al, 4.5% Ti, 1% Nb, and the rest was Ni. To obtain specimens with different initial grain sizes, solution heat treatment schemes were designed, namely 1150 °C/4 h, 1150 °C/6 h, 1150 °C/8 h, 1200 °C/2 h, 1200 °C/4 h followed by water quenching (WQ). WQ was to maintain the microstructure at high temperatures. To prevent Ni-Co-based superalloy from oxidation during the heat treatment process, the vacuum heat treatment method was used. The solution specimen was ground with 400–1000 grit SiC metallographic water sandpaper. The specimen was mechanically polished using a diamond metallographic spray polishing agent with a particle size of 0.5~2.5 μm until there were no obvious scratches on the surface of the specimen. Chemical etching was carried out in a mixed solution of 20 mL C_2_H_5_OH+20 mL HCl+3 gCuSO_4_, and the metallographic structure was observed using an OLYMPUS microscope (DSX 510, OLYMPUS, Tokyo, Japan).

**Figure 1:**
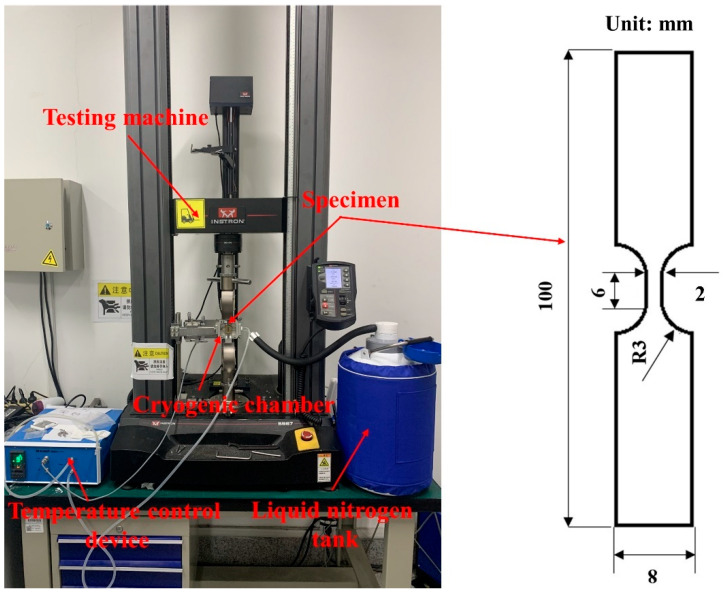
Ultra-low temperature tensile device.

The dog-bone-shaped micro-tensile specimens with a gauge width of 2 mm and a gauge length of 6 mm were prepared along the rolling direction (RD) using electrical discharge machining (EDM). A 30 kN electronic universal testing machine (Instron5967, INSTRON, Boston, MA, USA) was used to conduct the micro-tensile test, and the strain rate was set to 0.01 s^−1^. The ultra-low temperature tensile device included a cryogenic chamber, a temperature control device, and a liquid nitrogen tank, as shown in Figure 1. When the temperature stabilized at −180 °C, ultra-low temperature micro-tensile tests were performed. Each set of experiments was repeated 5 times to eliminate experimental errors. To study the deformation behavior of Ni-Co-based superalloys during the micro-tensile tests at room temperature and ultra-low temperature, tensile interruption tests were carried out. The tensile interruption specimens and fracture specimens were electrolytically polished in a mixed solution of 90% C_2_H_5_OH+10% perchloric acid. A scanning step size of 0.5 μm and a binning of 4 × 4 were used for EBSD (EDAX, Mahwah, NJ, USA) characterization. The fracture morphology of the specimens was characterized and analyzed using SEM (ZEISS, Oberkochen, Germany).

## 3. Results and Discussion

### 3.1. Microstructures

**Figure 2:**
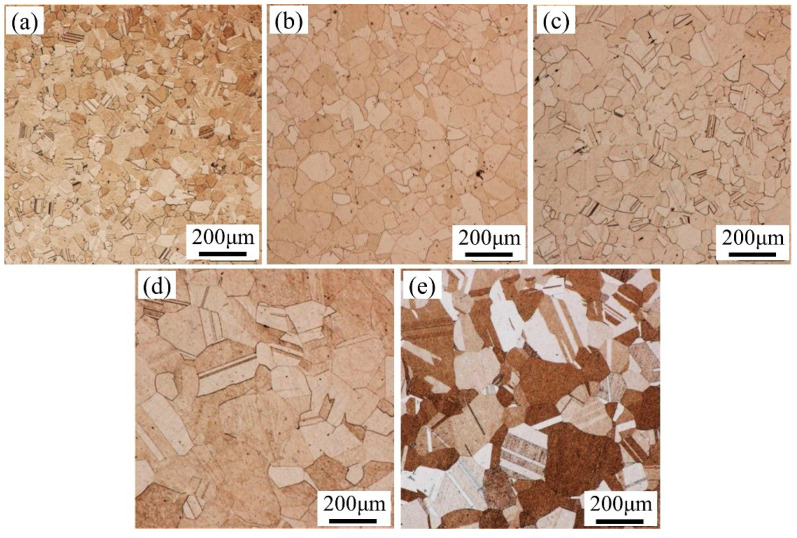
Metallographic structure of Ni-Co-based superalloy sheets after treatment with different solid solution regimes. (**a**) 1150 °C/4 h; (**b**) 1150 °C/6 h; (**c**) 1150 °C/8 h; (**d**) 1200 °C/2 h; (**e**) 1200 °C/4 h.

Figure 2 shows the metallographic structure of the Ni-Co-based superalloy specimen after it was subjected to high-temperature solution treatment. The metallographic structure after solution treatment was composed of evenly distributed equiaxed grains. The grain size gradually increased with increasing solution temperature. A certain amount of micron-scale annealing twins existed inside the grains. When the grain size was small, the material could be assumed to be isotropic, and the influence of grain orientation was not considered. However, as the grain size increased, the feature size of the specimen decreased, and the orientation of each grain began to play its role. The different orientations of each grain led to different corrosion resistances to metallographic corrosive agents. Grains that were not corrosion-resistant appeared dark (Figure 2e); otherwise, they appeared light. When the solution temperature reached a certain critical value, the resistance to grain boundary migration in the superalloy was significantly reduced, and the grains were easily coarsened, resulting in bigger grain sizes. The grain size of the solution specimen was measured using the cross-section method. The grain size and feature size are shown in Table 1. When the grain size increased from 23.3 μm to 84.8 μm, the feature size decreased from 8.6 to 2.4. It shows that the number of grains in the thickness direction of the specimen decreases significantly with increasing grain size.

**Figure 3:**
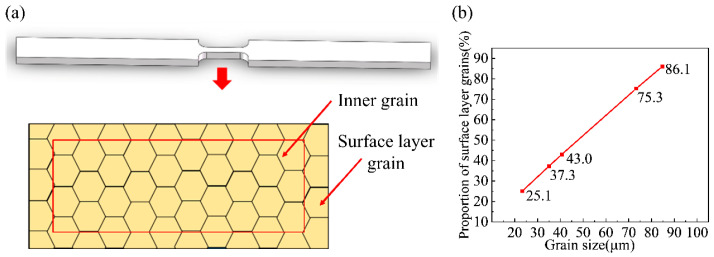
(**a**) Schematic diagram of the surface layer model; (**b**) surface layer grain proportion curve of specimens with different grain sizes of Ni-Co-based superalloy sheets.

At the mesoscopic scale, materials no longer behave as isotropic strain-hardening materials. The surface layer model is currently a widely recognized theory in the microforming field [2,45]. The surface layer model divides metal materials into surface layer grains with a softening effect and internal grains with strain hardening, as shown in Figure 3a. Controlling the grain size of the initial microstructure can change the proportion of surface grains and internal grains. According to Equation (1), the proportion of grains in the surface layer can be calculated as:

(1) $$\alpha =1-\frac{\left(t-2d)(W-2d\right)}{Wt}$$

where *α* represents the proportion of grains in the surface layer of the specimen; *t* represents the thickness of the specimen (μm); *W* represents the width of the specimen (μm); *d* represents the average grain size of the specimen (μm).

Figure 3b shows the surface layer grain proportion curves of specimens with different grain sizes. As the grain size increased, the proportion of surface layer grains increased. When the grain size was 23.3 μm, *α* was 25.1%. However, when the grain size was 84.8 μm, *α* reached 86.1%, which led to an obvious softening effect of flow stress.

### 3.2. Tensile Properties

**Figure 4:**
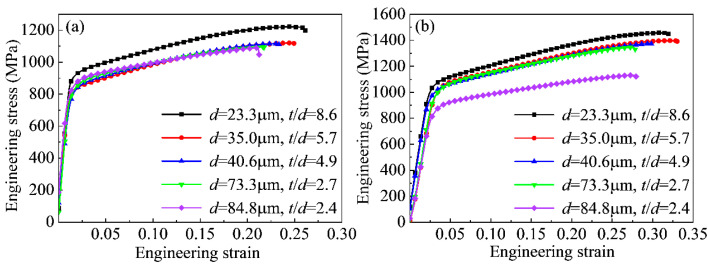
Engineering stress–strain curve of Ni-Co-based superalloy thin sheets (**a**) at room temperature; (**b**) at ultra-low temperature.

The engineering stress–strain curve has universal significance because the curve reflects various deformation processes such as plasticity, yield, and fracture that occur in the material under the action of external forces. Figure 4 shows the micro-tensile engineering stress–strain curves of the Ni-Co-based superalloy at room temperature and ultra-low temperatures. The engineering stress increased with the engineering strain under the same grain size due to work hardening. Additionally, the engineering stress gradually increased with decreasing grain size under the same engineering strain due to grain boundary strengthening. At the same deformation temperature, the stress of the specimen decreased significantly with decreasing *t*/*d*. When *t*/*d* decreased from 8.6 to 2.4, the tensile strength *σ*_b_ decreased from 1221 MPa to 1090 MPa, and the yield strength *σ*_s_ decreased from 793 MPa to 622 MPa at room temperature. When *t*/*d* decreased from 8.6 to 2.4, *σ*_b_ decreased from 1458 MPa to 1132 MPa, and *σ*_s_ decreased from 917 MPa to 730 MPa at ultra-low temperatures. The engineering stress of the micro-tensile specimen with a grain size of 84.8 μm increased with increasing engineering strain. However, the increasing degree of engineering stress was slightly lower compared to those of micro-tensile specimens with other grain sizes. This was because the surface layer grain deformation was less constrained due to the existence of the free surface, and dislocations were not easily accumulated in the surface layer grains, resulting in a reduction in the strain hardening ability of the surface layer grains and lower deformation resistance. At the same *t*/*d*, the stress of the specimen increased significantly with decreasing deformation temperature. It could be inferred that the ultra-low temperature was beneficial in improving the strength of the materials. The elongation of the alloy was better due to the matrix γ phase of the face-centered cubic (FCC) structure. When *t*/*d* decreased from 8.6 to 2.4, the elongation decreased from 0.26 to 0.21 at room temperature, and the elongation decreased from 0.31 to 0.28 at ultra-low temperatures. As the proportion of the surface layer grains increased, the number of grains contained in the thickness direction decreased, weakening the deformation coordination ability. Thus, the elongation of the Ni-Co-based superalloy decreased with decreasing *t*/*d*. At the same *t*/*d*, the elongation of the specimen increased significantly with decreasing deformation temperature. In summary, the strength and elongation of materials under the ultra-low temperature conditions were significantly better than those at room temperature. Therefore, ultra-low temperatures had a strengthening and plasticizing effect on the Ni-Co-based superalloys.

### 3.3. Plastic Deformation Mechanism

**Figure 5:**
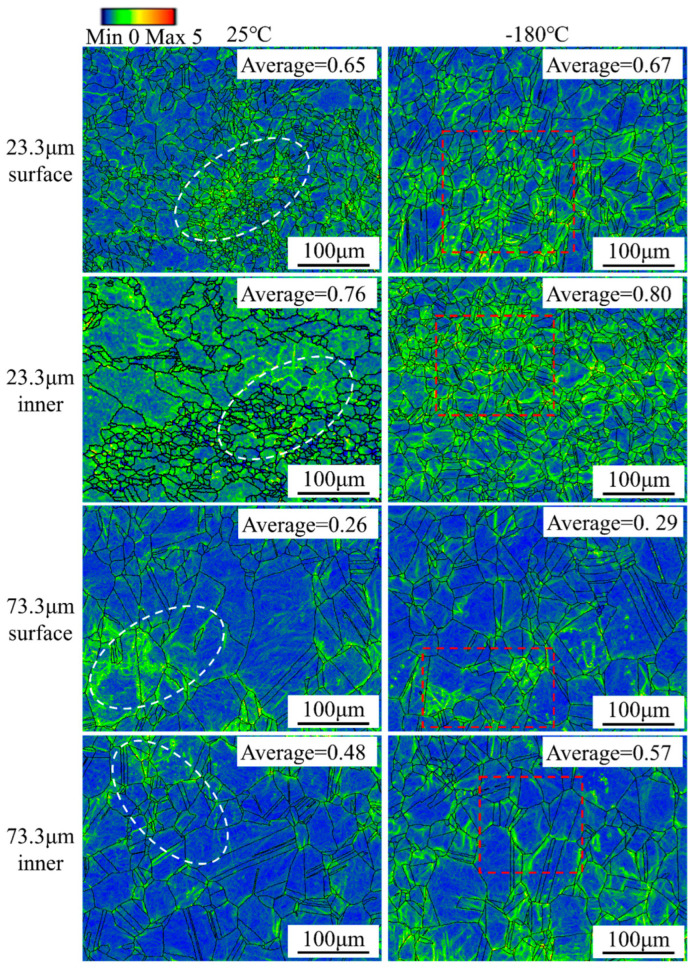
KAM distribution diagram of Ni-Co-based superalloy thin sheets at room temperature and ultra-low temperature when the strain was 0.19.

**Figure 6:**
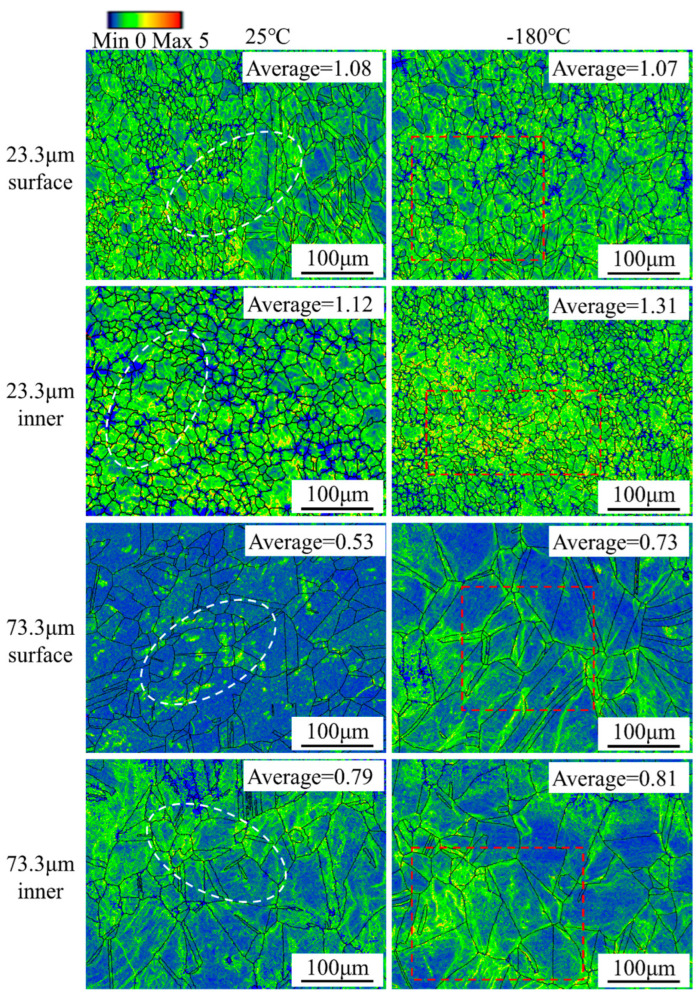
KAM distribution diagram of Ni-Co-based superalloy thin sheet fracture specimens at room temperature and ultra-low temperature.

Plastic strains can be characterized by local micro-orientations in the material [46]. KAM represents the local micro-orientations in the material. Figure 5 and Figure 6 show the KAM diagrams of the Ni-Co-based superalloy thin sheet specimens at room temperature and ultra-low temperatures when the strain was 0.19 and after the specimen fractured. The average KAM value can only be used to illustrate the overall uniformity of orientation distribution. As the grain size increased, the average KAM value of the Ni-Co-based superalloy thin sheet specimens decreased, and the distribution uniformity became worse. Local areas with higher KAM values appeared. Additionally, the average KAM values of the Ni-Co-based superalloy thin sheet specimens at ultra-low temperatures were slightly higher than those at room temperature. This shows that ultra-low temperatures helped to improve the plasticity of the alloy. Figure 5 and Figure 6 were mainly used to analyze the influence of grain size and deformation temperature on KAM value distribution, thereby revealing the influence mechanism of grain size and deformation temperature on deformation uniformity.

As shown in Figure 5, at room temperature, the average KAM values of the surface layer and internal grains of the specimen with a grain size of 23.3 μm were 0.65 and 0.76, respectively; the average KAM values of the surface layer and internal grains of the specimen with a grain size of 73.3 μm were 0.26 and 0.48, respectively. At ultra-low temperatures, the average KAM values of the surface and internal grains of the specimen with a grain size of 23.3 μm were 0.67 and 0.80, respectively; the average KAM values of the surface and internal grains of the specimen with a grain size of 73.3 μm were 0.29 and 0.57, respectively. The KAM value of the specimen with a grain size of 23.3 μm was relatively evenly distributed within the grain. The specimen with a grain size of 73.3 μm had a concentrated area of KAM value. As the grain size increased, the deformation ability of the alloy became worse. Additionally, compared with the deformation of internal grains, the deformation coordination ability of surface layer grains was poor. When there are only a few grains in the thickness of the specimen, the grains in the surface layer are less obstructed. Considering that the orientation of individual grains in the surface layer grains is randomly distributed, the inhomogeneous deformation of surface layer grains becomes more significant. Therefore, the average KAM value of the surface layer grains for the specimen with a grain size of 73.3 μm was the smallest.

When the grain size was small, the number of grains in the thickness direction of the specimen was large, and the deformation of the grains could be distributed to adjacent grains to coordinate the deformation, making the plastic deformation relatively homogeneous. As the grain size increased, the number of grains in the thickness direction of the specimen decreased, and the proportion of surface layer grains increased, which produced significant size effects. Therefore, as the proportion of the surface layer grains increased, the deformation coordination ability of materials weakened during the micro-tensile deformation process. At the same time, soft-oriented grains were easy to deform, while hard-oriented grains were difficult to deform. The material tended to follow the direction of minimum acting. The reduction in grains contained in the thickness direction of the thin sheet tended to form a “weak and weak” bond, causing strain concentration in the specimen and premature fracture.

As the deformation temperature increased, the KAM value of the Ni-Co-based superalloy specimen decreased, and the deformation ability decreased. When the strain was 0.19, obvious concentrated areas with larger KAM values appeared in the surface grains and internal grains of the specimen at room temperature. However, the areas with larger KAM values were more evenly distributed throughout the grain at ultra-low temperatures. The KAM value distribution of the fractured specimen in Figure 6 shows that the larger KAM values at room temperature were mainly concentrated at the grain boundaries. However, the larger KAM values at ultra-low temperatures were distributed at the grain boundaries and inside the grains. On the one hand, dislocation recovery was inhibited at ultra-low temperatures, causing the dislocation density to increase. On the other hand, as the deformation temperature decreased, the stacking fault energy of the alloy decreased and the width of the extended dislocation increased, causing the degree of dislocation accumulation to increase and the geometrically necessary dislocation density to increase. Thus, ultra-low temperature deformation improved the deformation homogenization of micro-tensile specimens.

The plastic deformation and ductile fracture at the mesoscopic scale are very different from those at the macroscopic scale. Surface roughening occurs during the plastic deformation of polycrystalline materials, which affects the subsequent plastic deformation behavior and formability of the material. The evolution of specimen surface roughness is mainly affected by the initial microstructure and deformation conditions. To explore the effects of grain size and deformation temperature on material flow behavior during micro-tensile deformation, the surface roughness was measured using a confocal laser scanning microscope (CLSM) (OLS3000, OLYMPUS, Tokyo, Japan) on the gauge length part of the specimen with a scanning step of 0.3 μm and an observation area of 640 μm × 640 μm.

**Figure 7:**
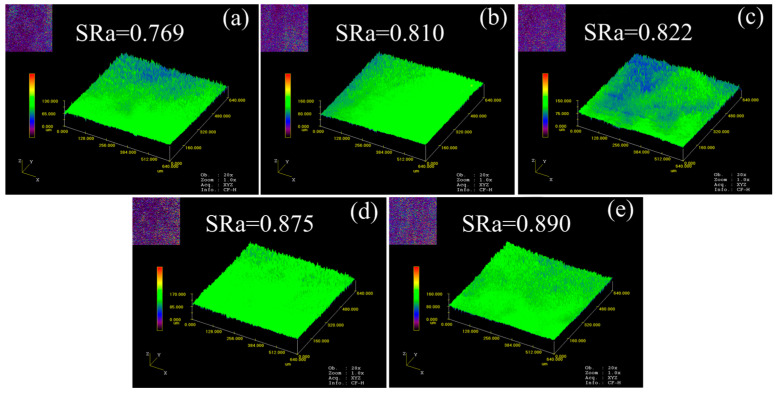
Surface roughness of micro-tensile specimens at room temperature. (**a**) *d* = 23.3 μm; (**b**) *d* = 35.0 μm; (**c**) *d* = 40.6 μm; (**d**) *d* = 73.3 μm; (**e**) *d* = 84.8 μm.

**Figure 8:**
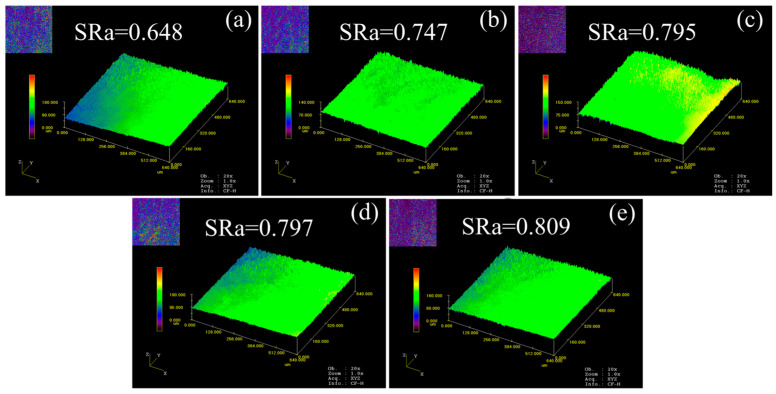
Surface roughness of micro-tensile specimens at ultra-low temperature. (**a**) *d* = 23.3 μm; (**b**) *d* = 35.0 μm; (**c**) *d* = 40.6 μm; (**d**) *d* = 73.3 μm; (**e**) *d* = 84.8 μm.

Figure 7 and Figure 8 show the surface roughness distribution of micro-tensile specimens at room temperature and ultra-low temperatures, respectively. “Peaks” and “valley” morphologies were observed on the surface of the specimen. When the grain size increased from 23.3 μm to 84.8 μm, the surface roughness of the specimen increased from 0.769 to 0.890 at room temperature and increased from 0.648 to 0.809 at ultra-low temperatures. The results show that the increase in grain size intensified the surface roughening of deformed specimens at the mesoscopic scale. Additionally, the differences in “peaks” and “valley” morphologies may be large in local areas of the specimen surface after deformation. However, SRa represents the average roughness of the smaller observation area. Thus, the variation is very little in surface roughness.

Differences in crystal orientation between adjacent grains led to surface roughening at the mesoscopic scale [47]. Polycrystalline materials can be viewed as a collection of many randomly distributed grains with different orientations. Therefore, the properties of the alloy are determined by the characteristics of each grain. At the macroscopic scale, there are many grains along the thickness of the material. Because the orientation and shape of these grains are randomly distributed, the alloy exhibits homogeneous properties. As the deformation scale is reduced to the micro/mesoscopic scale, there are only a few grains in the specimen thickness direction, and the grain size becomes closer to the feature size. When the grain size is small, the deformations of individual grains restrict each other, resulting in a homogeneous performance of the alloy. The grain boundary density decreases with increasing grain size. Considering that the orientation of individual grains is randomly distributed, the inhomogeneous deformation of surface layer grains becomes more significant. When there are only a few grains in the thickness of the specimen, the grains in the surface layer are less obstructed and easily deformed, and the roughening of the free surface causes strain concentration. The roughening of the free surface will cause strain localization in subsequent deformation, resulting in a reduction in the fracture strain.

Additionally, the surface roughness of specimens at ultra-low temperatures is lower than that at room temperature. This may be due to the fact that an ultra-low temperature reduces the dislocation activation energy and inhibits dislocation recovery, which increases the dislocation density within the grain. Compared with the deformation at room temperature, the deformation at ultra-low temperatures is borne more within the grain, thereby reducing the overall rotation of the grain. Thus, the deformation becomes more homogeneous, resulting in less surface roughening.

**Figure 9:**
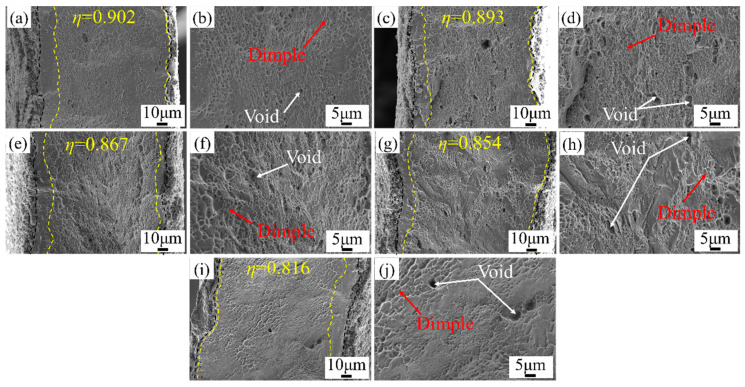
Fracture morphology of micro-tensile specimens at room temperature. (**a**,**b**) *d* = 23.3 μm; (**c**,**d**) *d* = 35.0 μm; (**e**,**f**) *d* = 40.6 μm; (**g**,**h**) *d* = 73.3 μm; (**i**,**j**) *d* = 84.8 μm.

**Figure 10:**
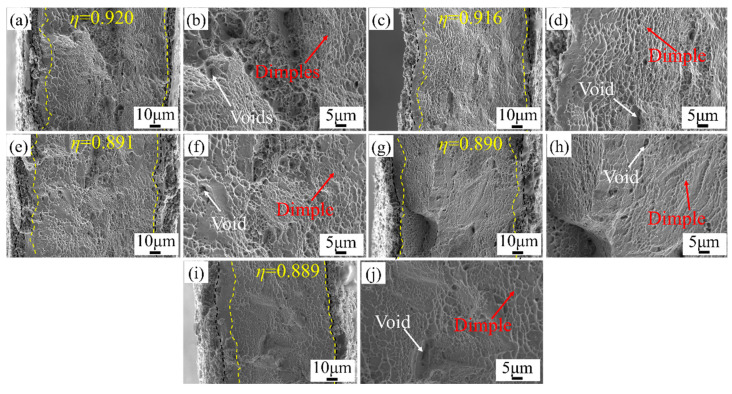
Fracture morphology of micro-tensile specimens at ultra-low temperature. (**a**,**b**) *d* = 23.3 μm; (**c**,**d**) *d* = 35.0 μm; (**e**,**f**) *d* = 40.6 μm; (**g**,**h**) *d* = 73.3 μm; (**i**,**j**) *d* = 84.8 μm.

Figure 9 and Figure 10 show the fracture morphology of micro-tensile specimens at room temperature and ultra-low temperatures. The necking occurred after the specimens were deformed, and the deformation in the thickness direction was inhomogeneous. The degree of necking at ultra-low temperatures was greater. The fracture surface was covered with a large number of equiaxed dimples. The dimples were traces left by voids nucleating, growing, and gathering on the fracture surface under the action of external forces. The dimples were caused by shear stress. The micro-tensile deformation of the Ni-Co-based superalloys was mainly micropore aggregation-type fractures. As the grain size increased, the equiaxed dimples on the fracture surface gradually evolved into shear dimples. Additionally, the number of dimples and voids decreased, and larger-sized dimples and voids appeared. Meng et al. [47] and Xu et al. [48] also obtained the same results when studying the effect of grain size on ductile fractures of pure copper sheets at the mesoscopic scale. Generally, the size and depth of dimples depend on the plastic deformation ability of the γ matrix and precipitated phases. There was almost no precipitate in the matrix γ phase after the high-temperature solution treatment. Thus, the plastic deformation ability of the matrix γ phase dominated the size and depth of dimples. The proportion of grain boundaries decreased with increasing grain size, reducing the coordinated deformation ability between adjacent grains. Therefore, the size and depth of dimples on the fracture surface increased with increasing grain size. The diameter of the large and deep dimples was about 5–7 μm, and the diameter of the small and shallow dimples was about 1–3 μm.

As shown in Figure 9, the strong interaction between dislocations and grain boundaries caused a large number of dislocations to accumulate at the grain boundaries during room temperature deformation, resulting in a reduction in deformation uniformity and easily leading to crack initiation. As the grain size increased from 23.3 μm to 84.8 μm, the fracture ductility area ratio *η* of the specimen decreased from 0.902 to 0.816, with a decrease rate of 9.5%. The increase in the proportion of the surface layer grains promoted the development of local necking and reduced the deformation uniformity.

As shown in Figure 10, compared with the fracture morphology at room temperature, the fracture surface consisted of a large number of deep dimples, which were evenly distributed, at ultra-low temperatures. This shows that the specimen had good plasticity at ultra-low temperatures. As the grain size increased from 23.3 μm to 84.8 μm, *η* decreased from 0.920 to 0.889 with a decrease rate of 3.4%. The difference in deformation between the surface grains and the internal grains was reduced, indicating that the specimen deformed more homogeneously at ultra-low temperatures. High local misorientation was mainly concentrated inside the grains at ultra-low temperatures, and the storage capacity of dislocations was enhanced. The weakening of strain concentration at grain boundaries resulted in a delayed onset of cracks. Thus, ultra-low temperatures enhanced the ductility of Ni-Co-based superalloys.

After high-temperature solution treatment, there were no second-phase particles in the matrix γ phase. The deformation of surface layer grains was less constrained due to the presence of free surfaces. The surface layer grains were easy to rotate to promote the movement of dislocations, thereby reducing the flow stress and hindering the occurrence of void nucleation in the surface layer grains. The rotation of the grains caused uneven surface deformation. The rotation of grains was considered as a possible explanation for surface roughening during plastic deformation [49,50]. The roughening of the free surface during deformation caused strain localization in subsequent deformations, resulting in a reduction in the fracture strain. The (111)<110> slip system was relatively easy to activate under the action of shear stress due to the FCC structure of the matrix γ phase of the Ni-Co-based superalloy. The plastic deformation of the internal grains was limited by the surrounding grains. The movement of dislocations in the internal grains was restricted by grain boundaries. Dislocations were prone to accumulate at the grain boundaries of internal grains, forming dislocation accumulation or entangling into a dislocation network, thereby causing the stress concentration at grain boundaries. When the stress concentration reached a certain critical value, voids initiated at the grain boundaries. As the grain size increased, the coordinated deformation ability between different grains decreased. Slip bands were susceptible to single-grain orientation and properties, with strain localization occurring at individual grain boundaries. As the grain size increased, the ductility area ratio of the fracture surface decreased. When the number of grains present in the thickness direction of the specimen was small, the deformation tended to become localized in the early stage. As the plastic deformation continued, the intragranular plastic deformation intensified, and dimples began to nucleate inside the grains. Under the action of shear stress, low-stress triaxiality (*η*) [51,52] caused dimple growth and expansion, leading to the generation of microcracks. The stress concentration caused by the accumulation of dimples could lead to accelerated plastic deformation between adjacent dimples, resulting in severe strain localization. Therefore, the coupled effects of surface roughening caused by free surface grains and hindered dislocation movement induced by grain boundaries during plastic deformation resulted in strain localization, causing fracture failure of the Ni-Co-based superalloys.

## 4. Conclusions

This study carried out micro-tensile tests on Ni-Co-based superalloy thin sheets. The effects of grain size and deformation temperature on micro-tensile mechanical properties and microstructure evolution were analyzed, and the micro-tensile fracture mechanism was revealed. The main conclusions are as follows:

(1) Different grain sizes were controlled with high-temperature solution treatment, and the average grain size of the Ni-Co-based superalloy increased from 23.3 μm to 84.8 μm and the feature size decreased from 8.6 to 2.4 with increasing solution temperature.
(2) The flow stress decreased significantly with increasing grain size and increased significantly with decreasing deformation temperature. When *t*/*d* decreased from 8.6 to 2.4, the tensile strength *σ*_b_ decreased from 1221 MPa to 1090 MPa, the yield strength *σ*_s_ decreased from 793 MPa to 622 MPa, and the elongation decreased from 0.26 to 0.21 at room temperature. When *t*/*d* decreased from 8.6 to 2.4, *σ*_b_ decreased from 1458 MPa to 1132 MPa, *σ*_s_ decreased from 917 MPa to 730 MPa, and the elongation decreased from 0.31 to 0.28 at ultra-low temperatures.
(3) The surface roughness of the specimen increased with decreasing *t*/*d* and decreased with decreasing deformation temperature. Ultra-low temperatures enhanced the plastic deformation ability of the alloy. When *t*/*d* decreased from 8.6 to 2.4, the surface roughness of the specimen increased from 0.769 to 0.890 at room temperature and increased from 0.648 to 0.809 at ultra-low temperatures.
(4) The coupled effects of surface roughening caused by free surface grains and hindered dislocation movement induced by grain boundaries during plastic deformation resulted in strain localization, causing fracture failure of the Ni-Co-based superalloys.

## Figures and Tables

**Table 1:** Grain size and feature size of Ni-Co-based superalloy specimens under various solution treatment regimes.

Heat Treatment Regime	1150 °C/4 h	1150 °C/6 h	1150 °C/8 h	1200 °C/2 h	1200 °C/4 h
*d* (μm)	23.3	35.0	40.6	73.3	84.8
*t/d*	8.6	5.7	4.9	2.7	2.4

## Data Availability

Data are available upon request due to privacy restrictions.
